# Sutureless Repair for Iliac Vein Bleeding Using an Elastomeric Sealant

**DOI:** 10.7759/cureus.57074

**Published:** 2024-03-27

**Authors:** Yoshinori Nakahara, Takeyuki Kanemura, Motoharu Shimozawa, Shuhei Kawamoto, Toshiya Fukushima, Kazuki Morooka

**Affiliations:** 1 Cardiovascular Surgery, IMS Katsushika Heart Center, Tokyo, JPN; 2 Cardiothoracic Surgery, IMS Katsushika Heart Center, Tokyo, JPN

**Keywords:** hemostatic agent, iliac vein injury, y-grafting, sutureless repair technique, venous bleeding

## Abstract

Addressing venous bleeding is a frequent complication of vascular and abdominopelvic surgeries. We present a novel sutureless repair technique using Hydrofit^Ⓡ^ (Terumo, Tokyo, Japan), an elastomeric sealant. In a patient experiencing common iliac vein bleeding during abdominal aortic aneurysm surgery, this technique successfully achieved complete hemostasis. By applying the elastomeric sealant with an autologous arterial patch to the bleeding site, we demonstrate the simplicity and effectiveness of sutureless hemostasis for venous bleeding.

## Introduction

Iatrogenic bleeding from veins like the vena cava is a well-known complication of vascular, abdominal, and pelvic surgeries. While major injuries are uncommon, their consequences can be devastating [1-3]. For traumatic iliac injury, mortality rates vary widely in the literature, ranging from 25% to 80% [4,5], reflecting the severity and complexity of these injuries. Notably, iliac vascular injuries are predominantly caused by penetrating trauma, which tends to result in more severe outcomes compared to blunt trauma. A significant challenge in evaluating the literature is the tendency to amalgamate data from both blunt and penetrating traumas, including cases with concurrent injuries. This approach has likely led to an overestimation of mortality and morbidity rates for isolated iliac vascular injuries due to the rarity of these events and their frequent association with additional traumas. Hence, the precise impact and prognostic indicators of isolated iliac vascular injuries remain elusive.

In cases of venous injury, the inherent fragility of venous walls poses a substantial challenge for surgeons during sutured repair of venous injuries. Conversely, sutureless repair using hemostatic agents often proves less effective for major bleeding, explaining the limited number of previous reports. This report presents a case of successful sutureless repair using Hydrofit^Ⓡ^ (Terumo, Tokyo, Japan) for major bleeding from the common iliac vein.

## Technical report

**Figure 1:**
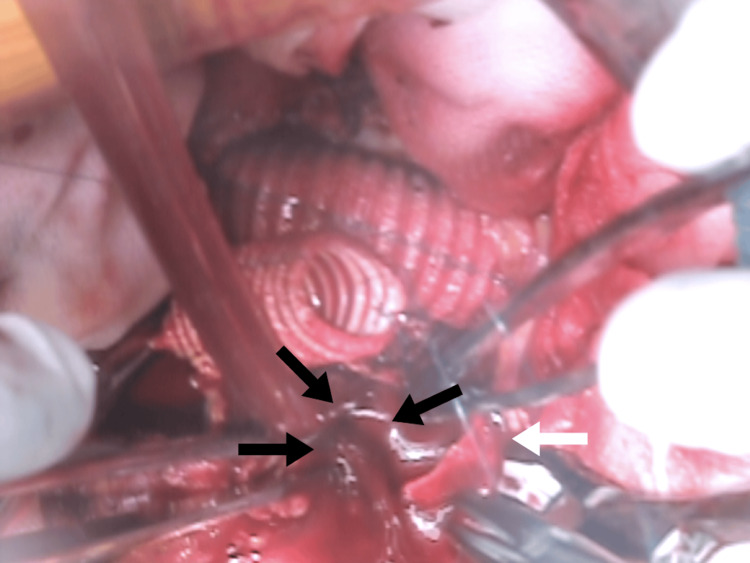
Bleeding from the right common iliac vein During anastomosis of the right common iliac artery (white arrow), the right common iliac vein was accidentally injured (black arrows).

**Figure 2:**
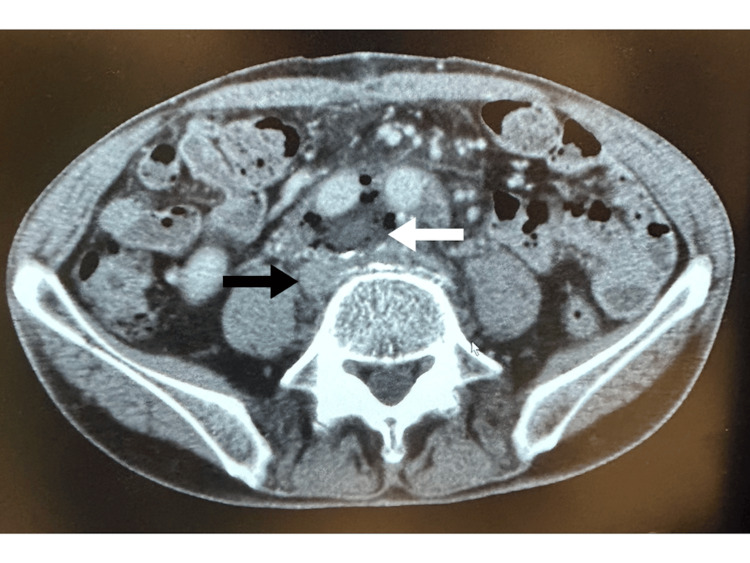
Post-operative computed tomography Post-operative computed tomography scan shows no bleeding or hematoma around the repaired right common iliac vein (black arrow). The white arrow points to the applied Hydrofit^Ⓡ^, arterial patch, and Surgicel^Ⓡ^.

A 77-year-old man with an abdominal aortic aneurysm, measuring 60 mm in maximum short diameter, underwent artificial vessel replacement. Using a midline abdominal incision, systemically heparinisation was administered. The infrarenal abdominal aorta and common iliac arteries were bilaterally clamped. An incision was made in the aorta, and a Y-shaped artificial vessel and 4-0 polypropylene were used to continuously suture the proximal anastomosis. During the right common iliac artery anastomosis, the right common iliac vein surface sustained accidental injury (Figure 1). An initial attempt at repair with a U-suture, 5-0 polypropylene, and a pledget caused further tearing and increased bleeding. Consequently, sutureless repair with Hydrofit^Ⓡ^ was employed. An iliac arterial wall patch was harvested, and Hydrofit^Ⓡ^ along with the patch was applied to the bleeding site. Manual compression for two minutes achieved complete hemostasis (Video 1). The right and left common iliac arteries were anastomosed, and the procedure was completed. Extubation and transfer to the intensive care unit followed. The patient was discharged on postoperative day six and is recovering well in an outpatient setting. Contrast-enhanced computed tomography on the fifth postoperative day confirmed successful hemostasis, showing no bleeding or hematoma retention in the repaired right common iliac vein (Figure 2) and ruling out pulmonary embolism.

**Video 1:** Sutureless repair for iliac vein bleeding using an elastomeric sealant Surgical movie. Hydrofit^Ⓡ^ and arterial patch were applied to the bleeding area, achieving complete hemostasis after two minutes of manual compression.

## Discussion

We present a successful case of sutureless repair for massive bleeding from the iliac vein during abdominal aortic surgery. Hemostatic methods for venous injuries fall into two categories: sutured and sutureless repairs. However, fragile vein walls make sutured repairs prone to further bleeding, a particularly catastrophic concern in abdominal aortic aneurysms, gastrointestinal, and obstetrics and gynecology surgeries where cardiopulmonary bypass is not used. Therefore, successful sutureless repair for major venous bleeding holds immense promise for better surgical outcomes.

Hydrofit^Ⓡ ^is a sealant comprised of reactive isocyanate groups and a fluorine-containing polyether polyurethane prepolymer. It reacts rapidly with tissue water to form a robust adhesive film [6]. Unlike popular options like fibrin glue or TacoSeal^Ⓡ^ (CSL Behring, King of Prussia, USA), Hydrofit^Ⓡ^ thrives in moist environments and boasts precise application thanks to its viscosity, preventing unwanted spread into vessels [7]. Moreover, Hydrofit^Ⓡ^ remains effective even with coagulopathy or heparinisation, as it bypasses the blood coagulation cascade altogether.

The primary strength of Hydrofit^Ⓡ^ lies in its potent hemostatic effect. While evidence for Hydrofit^Ⓡ^’s intraoperative hemostasis is burgeoning, its long-term effects require further scrutiny. Previous studies showcase successful sutureless repair using Hydrofit^Ⓡ^ in a pig model with an inferior vena cava incision [7] and a case of coronary sinus rupture in cardiac surgery [8]. An in vitro and in vivo study also reported its efficacy in achieving hemostasis in rats, when combined with Surgicel^Ⓡ^ (Ethicon, Inc., Somerville, USA) [9].

The primary strength of Hydrofit^Ⓡ^ lies in its potent hemostatic effect. While reports exist for Hydrofit^Ⓡ^’s success in arterial hemorrhages, like a ruptured left ventricle [10], remote-stage results remain unclear. Pseudoaneurysms have been reported after sutureless repair for arterial pressure bleeds [11]. However, venous bleeding carries a lower risk of recurrence due to its lower pressure, as confirmed by our safety report two years postoperatively [7]. In this case, we strategically combined Hydrofit^Ⓡ^ with an arterial patch, ensuring that even if Hydrofit^Ⓡ^ loses its effect, the patch continues to prevent further bleeding. In addition to using bovine pericardium and arterial patches, we also believe that venous patches and fascia could serve as suitable substitutes. Long-term studies on sutureless repair for venous hemorrhages are crucial for definitive conclusions.

## Conclusions

Our case demonstrates the effectiveness of sutureless repair with Hydrofit^Ⓡ^ for managing massive bleeding from the common iliac vein during abdominal aortic replacement. Hydrofit^Ⓡ^ is a novel tissue adhesive formed by a reaction between a copolymer of polyethylene glycol and polypropylene glycol with fluorinated hexamethylene diisocyanate. Its formulation reduces the risk of tissue damage and enhances usability in moist environments, distinguishing it from other surgical adhesives through its mechanism of water absorption.
